# A graph-theoretic framework for quantitative analysis of angiogenic networks

**DOI:** 10.1186/s13040-025-00478-1

**Published:** 2025-10-02

**Authors:** Goodluck Okoro, Pawel Wityk, Michael B. Nelappana, Karl A. Jackiewicz, Veronica Z. Kucharczyk, Annie Tigranyan, Catherine C. Applegate, Iwona T. Dobrucki, Lawrence W. Dobrucki

**Affiliations:** 1 Department of Bioengineering, University of Illinois at Urbana−Champaign, Urbana, IL USA; 2 Beckman Institute for Advanced Science and Technology, University of Illinois at Urbana- Champaign, Urbana, IL USA; 3 Department of Biomedical and Translational Sciences, Carle-Illinois College of Medicine, University of Illinois at Urbana-Champaign, Urbana, IL 61853 USA; 4 Department of Chemical and Biomolecular Engineering, University of Illinois at Urbana−Champaign, Urbana, IL USA; 5 Carle Illinois College of Medicine, University of Illinois at Urbana-Champaign, Urbana, IL USA; 6 Cancer Center at Illinois, University of Illinois at Urbana-Champaign, Urbana, IL USA; 7 Academy of Medical and Social Applied Sciences, Elblag, Poland; 8 Division of Medical Laboratory Diagnostics - Fahrenheit Biobank BBMRI.pl, Medical University of Gdansk, Gdansk, Poland; 9 University of Illinois at Urbana-Champaign, 405 N. Mathews Ave, MC-251, Urbana, IL 61801 USA

**Keywords:** Angiogenesis, Tube formation assay, Graph theory, Network morphology, Spatial heterogeneity

## Abstract

The endothelial tube formation assay is an established in vitro model for evaluating angiogenesis. Although widely used, quantification of angiogenic behavior in such assays remains semi-empirical and often lacks spatial, topological, and structural context. Here, we present a graph-theoretic framework to quantify network morphology, temporal dynamics, and spatial heterogeneity in tube formation assays. We simulated two distinct angiogenic network morphologies using human umbilical vein endothelial cells (HUVECs) seeded at two densities and imaged at 2, 4, and 18 h post-seeding. Skeletonized images were converted to mathematical graphs from which 11 graph-based metrics were extracted. This framework captured both morphological differences and temporal progression. Sparse networks exhibited significantly higher *average node degree* (*p* = 0.00079), *clustering coefficient* (*p* = 0.00109), and *tortuosity* (*p* = 0.0171), whereas dense networks showed greater *node and edges counts* (*p* = 0.00109). Over time, networks evolved from fragmented forms at 2 h to integrated structures at 18 h, as reflected by increased *largest component size* (*p* = 0.00216), *connectivity index* (*p* = 0.00216), and *efficiency* (*p* = 0.0152). ROC AUC analysis revealed that metrics such as *average degree* (AUC = 0.98) and *clustering coefficient* (AUC = 0.96) effectively distinguished between sparse and dense morphologies, while component-based metrics perfectly separated 2- and 18-hour networks (AUC = 1.00). Radial zone analysis revealed that vascular distribution becomes more compartmentalized over time, with increasing standard deviation and coefficient of variation. This approach provides a sensitive and scalable method for quantifying angiogenic dynamics, offering insight into both therapeutic efficacy and disease-related vascular remodeling.

**Supplementary Information:**

The online version contains supplementary material available at 10.1186/s13040-025-00478-1.

## Introduction

Angiogenesis is the process of forming new blood vessels from pre-existing vasculature, which is a critical component of tissue development, wound healing, and pathological conditions such as cancer and peripheral arterial disease [1–3] In vitro tube formation assays using endothelial cells remain a widely used method to assess angiogenic potential under various stimuli or therapeutic interventions [4–6]. Despite their prevalence, the quantification of angiogenic behavior in such assays remains semi-empirical and often lacks spatial, topological, and structural context [7].

Conventional image analysis methods typically measure parameters such as total tubule length, number of nodes, and junction density, often relying on binarized images and object detection algorithms [7, 8] Although quite useful for evaluating gross morphological traits, such approaches treat the vascular network as a collection of disconnected features rather than as an integrated system, lacking the capacity to evaluate how these features interconnect or evolve as a coordinated system. As a result, they fail to capture the underlying topological and organizational properties that define vascular connectivity and function. To overcome these limitations, we propose a graph-theoretic framework that transforms skeletonized images of endothelial networks into mathematical graphs, enabling a more rigorous and interpretable analysis of structural features and temporal patterns.

Graph theory provides a rich toolbox for characterizing the connectivity, complexity, and robustness of biological networks [9–12]. By representing vessel-like structures as nodes (junctions or pixels) and edges (connections), we can compute global and local metrics that reflect the efficiency, redundancy, and topology of the angiogenic network. These include *average degree*,* clustering coefficient*,* shortest path lengths*,* global efficiency*, and *tortuosity*, all of which have biological relevance in vascular remodeling and perfusion context [13–15]. Additionally, we introduced a radial zone analysis method centered on a biologically meaningful origin (e.g., geometric image center) to spatially profile how vessel density and connectivity distribute across concentric layers. This allows for the capture of spatial heterogeneity, which is often overlooked in traditional analyses. Finally, to enable robust comparisons across samples and conditions, we introduce a new metric named *“connectivity index”*, which quantifies how well-connected the network is, and it reflects overall network quality and complexity.

The proposed framework is implemented as a reproducible pipeline using open-source libraries in Python. It requires no manual annotation and applies to any tube formation image that can be processed to a binary skeleton. Our method significantly enhances the quantitative rigor of angiogenesis assays and can serve as a valuable tool for both mechanistic studies and high-throughput screening of pro-angiogenic therapeutics.

## Materials and methods

### HUVEC culture and tube formation assay

Human umbilical vein endothelial cells (HUVECs) were cultured in endothelial cell growth medium (ECGM, C-22010, Sigma-Aldrich) under standard conditions (37 °C, 5% CO₂). For the tube formation assay, 300 µL of Matrigel (Corning) was used to coat the wells of a 6-well plate, providing a reconstituted basement membrane matrix. To simulate two distinct angiogenic network morphologies and to evaluate how the framework can accurately distinguish different network morphologies, we used the initial cell-seeding density to influence network formation and stability over time. Two distinct seeding densities were used: a sparse condition with 5,000 HUVECs per well and a dense condition with 15,000 HUVECs per well. This design enabled us to simulate biologically driven alterations in network branching, connectivity, and spatial organization. Following seeding, cells were incubated without disturbance to promote tubulogenesis. Brightfield images were acquired using EVOS XL Core microscope at 2, 4, and 18 h post-seeding to capture the temporal dynamics of tubule formation. Each condition and timepoint included six biological replicates (*n* = 6). Acquired images were processed and quantitatively analyzed using the graph-theoretic pipeline detailed in the following sections. In addition to the graph-theoretical analysis, all images were also analyzed using the Angiogenesis Analyzer plugin in ImageJ, a widely used tool for quantitative assessment of in vitro vascular structures [8, 16]. This analysis extracted 20 standard morphometric metrics, including measurements of segment length, mesh area, number of junctions, and total branching length, among others. A full description of each metric is detailed here [8].

### Image acquisition and preprocessing

**Fig. 1:**
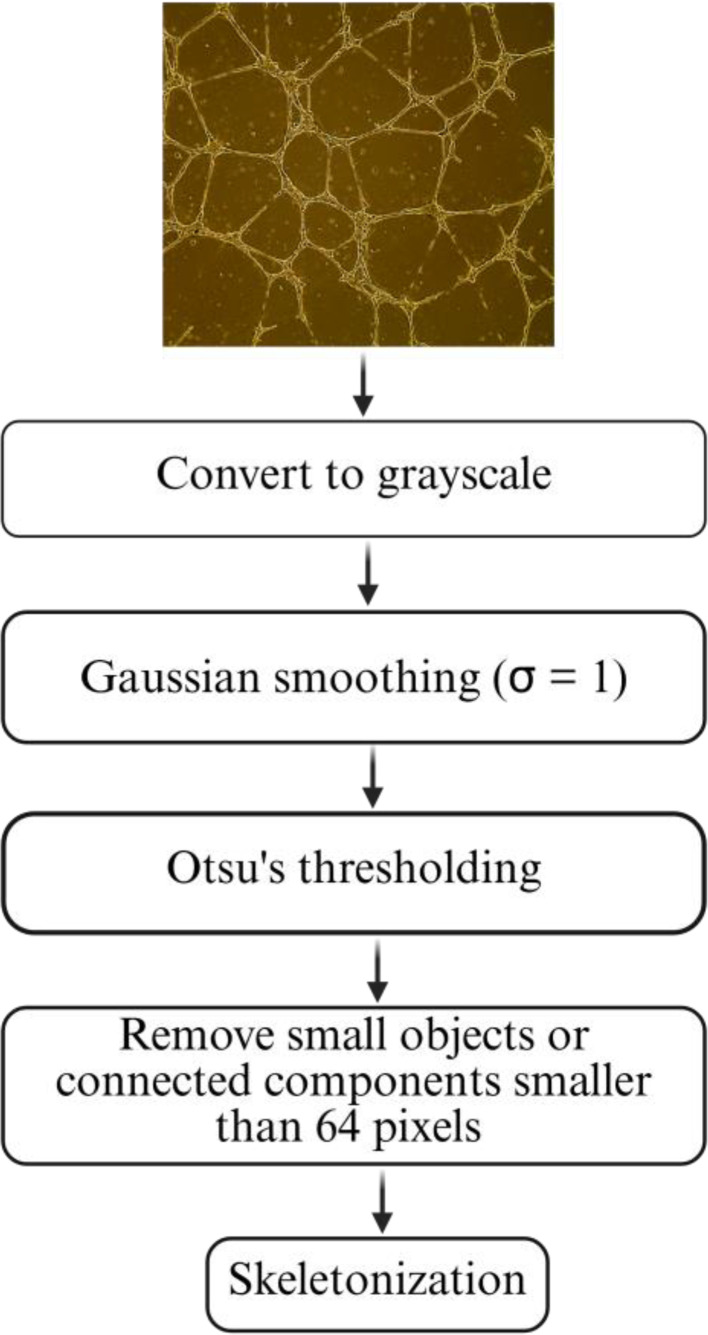
Overview of image preprocessing workflow

In this study, we have analyzed microscopy images from in vitro tube formation assays. After incubation, tubule structures were imaged using brightfield microscopy. All images were saved in JPEG format at full resolution to preserve morphological details. All image analyses and graph computations were performed using Python (v3.10.9). The following open-source libraries were employed; scikit-image for image processing (filtering, thresholding, and skeletonization), NetworkX for graph construction and metric computation, matplotlib for visualization, scipy and numpy for numerical operations [17–21]. To standardize image inputs and enhance contrast, each image underwent a series of preprocessing steps designed to extract vascular structures accurately while minimizing noise and artifacts. First, color images were converted to grayscale. Next, Gaussian smoothing with a standard deviation (σ ≈ 1) was applied to reduce high-frequency noise [22]. The smoothed image was then binarized using Otsu’s thresholding method to differentiate foreground tubule structures from the background [23, 24]. To eliminate small artifacts that could introduce false graph components, connected components smaller than 64 pixels were removed. This preprocessing pipeline ensured that only meaningful vascular features were retained for subsequent graph analysis (Fig. 1).

### Skeletonization and pixel connectivity

Following image binarization and artifact removal, the vascular structures were subjected to skeletonization which is a process that reduces objects in a binary image to their 1-pixel-wide centerlines while preserving their topology and connectivity [25] This transformation enables an efficient representation of the vascular network for downstream graph construction. Skeletonization was performed using the *skeletonize* function from the *skimage.morphology* module [21]. This algorithm applies iterative morphological thinning to reduce each foreground object to its medial axis. The result is a minimal representation where every retained pixel is topologically critical for maintaining the structure of the original network. To prepare the skeleton for graph-theoretic analysis, we identified all foreground pixels (skeleton pixels) and their spatial relationships. Each skeleton pixel was treated as a potential graph node, and its connectivity was determined by examining the eight neighboring pixels in its 3 × 3 neighborhood (8-connectivity).

Specifically, for each pixel, $\:p=(x,y)$, the following directional offsets were used to inspect neighbors:

1 $$\:{\mathcal{N}}_{8\:}\left(p\right)=\left\{\left(x+i,\:y+j\right)\:\right|\:i,\:j\:\in\:\left\{-1,\:0,\:1\right\},\:\left(i,j\right)\ne\:(0,\:0)\}$$

A connection (an edge) was established between pixel $$\:p$$ and each neighbor $$\:q\:\in\:\:$$$$\:{\mathcal{N}}_{8\:}\left(p\right)$$ that also belonged to the skeleton. The Euclidean distance $$\:d(p,\:q)$$ between two connected pixels was used as the weight for the corresponding edge:

2 $$\:d\left(p,q\right)=\:\sqrt{{({x}_{p}-{x}_{q})}^{2}+{({y}_{p}-{y}_{q})}^{2}}$$

In practice, because skeleton pixels can lie diagonally or adjacently, distances were typically either 1 for direct neighbors or $\:\surd\:2$ for diagonal neighbors. The pixel-level connectivity serves as the foundation for transforming the skeleton into a mathematical graph. We constructed an adjacency matrix representing pixel-level interactions, which was later used to construct a graph object by systematically evaluating the local neighborhood of each skeleton pixel.

### Graph construction from skeletons

**Fig. 2:**
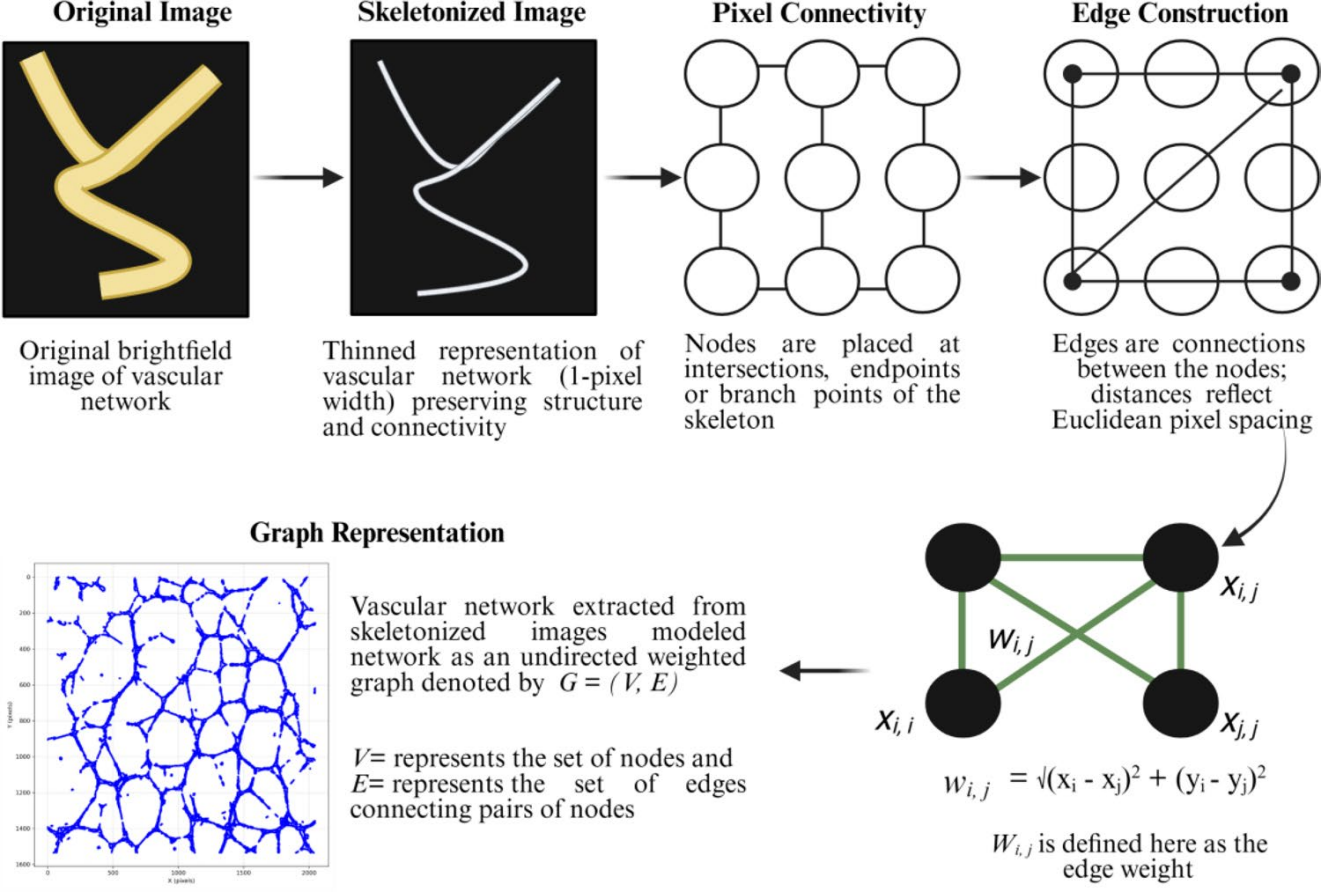
Overview of graph-based image quantification pipeline. (Created with BioRender.com)

After skeletonization, the resulting image consists of a set of 1-pixel-wide lines that represent the centerlines of vascular structures (Fig. 2). These pixels are treated as discrete spatial units from which a graph is constructed. Let the set of skeleton pixels be denoted as:

3 $$\:P=\left\{{p}_{1},\:{p}_{2},\dots\:..,{p}_{n}\right\},\:\text{w}\text{h}\text{e}\text{r}\text{e}\:{p}_{i}=({x}_{i},\:{y}_{i})$$

Each pixel $\:{p}_{i}$ becomes a node$\:\:{v}_{i}$ in an undirected graph $\:G=(V,E)$, where *V* is the set of nodes and *E* the set of edges. Two pixels $\:{p}_{i}$ and $\:{p}_{j}$ are connected if the Euclidean distance between them is less than or equal to $\:\surd\:2$ which captures direct 8-connectivity including diagonal neighbors on a 2D grid. In other words, an edge $\:{e}_{ij}$ exists between nodes $\:{v}_{i}$ and $\:{v}_{j}$ if:

4 $$\:{e}_{ij}\:\in\:E\:\:if\:\sqrt{{({x}_{i}-{x}_{j})}^{2}+{({y}_{i}-{y}_{j})}^{2}}\:\le\:\surd\:2$$

Each edge is weighted using the Euclidean distance between the corresponding pixel coordinates as shown in Equation (2). This process produces a weighted, undirected graph that encodes both the structure and spatial geometry of the network. All edges capture local connectivity, and all node positions are preserved as attributes for use in geometric and topological analyses in subsequent steps. For computational efficiency, the pairwise distance $\:D\in\:{\mathbb{R}}^{n\times\:n}$ are calculated only once, and edges are added based on the distance threshold condition. The final graph is sparse, with each node connected only to its immediate spatial neighbors.

### Computation of graph metrics

Once a graph is constructed from the skeletonized tubule formation image, we computed a set of 11 global and local graph metrics that quantitatively describe the structural and functional properties of the angiogenic network. Each metric is chosen for its biological relevance and interpretability in the context of vascular remodeling. Overall, the final graph $\:G=(V,E)$, where *V* is the set of nodes and *E* the set of edges represents the extracted endothelial network (Table 1).

**Table 1:** Summary of graph-theoretic metrics used, including definitions and their relevance to angiogenic network analysis

Graph Metrics	Description	Biological Significance
Number of Nodes	The count of all distinct pixels or points forming part of the vascular structure [26]	It indicates the complexity of the vascular structure. A higher node count suggests increased branching and sprouting
Number of Edges	The total number of direct connections between these nodes, as defined by their neighborhood (8-connected pixels) [12]	Describes how well-connected each vessel junction is. Higher values imply better-integrated vascular structures.
Average Node Degree	The degree of a node is the number of direct connections (edges) it has to other nodes [27]. This gives an idea of how well-connected the typical node is in the network. The average degree is defined as: $\>\langle k\rangle = \>{1 \over {\left| V \right|}}\>\sum\limits_{{\>_{v \in \>V}}} {\rm{deg}} (v)$	This quantifies the extent of local branching within the vascular network. It provides insight into whether angiogenic growth is random and proliferative (immature) or ordered and efficient (mature).
Average Clustering Coefficient	The clustering coefficient measures how likely it is that a node’s neighbors are connected to each other [28] It essentially captures local clustering or triangle formation. Mathematically, clustering coefficient can be calculated thus: For a node $\:v$ with $\:{k}_{v}$ neighbors, $\:{C}_{V}=\:\frac{{2T}_{v}}{{k}_{v}-(\:{k}_{v}-1)}$ Where $\:{T}_{v}$ is the number of triangles through node $\:v$. The average clustering coefficient is then: $\:C=\:\frac{1}{\left|V\right|}\sum\limits_{v\in\:V}{C}_{v}$	Represents active local vessel looping, branching or mesh-like organization.
Global Efficiency	[This is defined as the average inverse shortest path length between all node pairs: $\:{E}_{global}=\:\frac{1}{\left|V\right|(\left|V\right|-1)}\sum\limits_{i\ne\:j}\frac{1}{{d}_{ij}}$ Here, $\:{d}_{ij}$ is the shortest path length between nodes $\:i$ and $\:j$. Higher efficiency means shorter, more direct paths between nodes. This is desirable in perfused capillary structures.	Describes how efficiently flow or communication can be exchanged over the network [29]. High efficiency suggests that oxygen and nutrient transport through the network is optimized.
Betweenness Centrality	This metric quantifies how often a node lies on the shortest path between other pairs of nodes [30]. For node $\:v$ it is defined as: $\:BC\left(v\right)=\:\sum\limits_{s\ne\:v\ne\:t}\frac{{\sigma\:}_{st}\left(v\right)}{{\sigma\:}_{st}}$ Where, $\:{\sigma\:}_{st}$ is the total number of shortest paths between nodes $\:s$ and $\:t,\:$and $\:{\sigma\:}_{st}\left(v\right)$ is the number of those paths that pass through $\:v$. We report the average betweenness centrality across all nodes as: $\:Avg\:BC=\:\frac{1}{\left|V\right|}\sum\limits_{v\in\:V}BC\left(v\right)$	Highlights key transit hubs for flow. This can be interpreted thus: Nodes with high betweenness are critical for flow distribution, acting like hubs or bridges in the network.
Tortuosity	Tortuosity measures how winding or indirect a path is between two nodes compared to the straight-line (Euclidean) distance [31] For two nodes $\:i$ and $\:j$, tortuosity $\:{T}_{ij}$ is calculated as: $\:{T}_{ij}=\frac{{L}_{ij}}{{D}_{ij}}$ Where $\:{L}_{ij}$ is the shortest path length between $\:i$ and $\:j$ while $\:{D}_{ij}$ is the Euclidean distance between $\:i$ and $\:j$. We have calculated Average tortuosity over a subset of randomly sampled node pairs to manage computation time.	In this context, higher tortuosity indicates more convoluted vessel paths, which may reflect immature or inefficient tubes. However, broadly speaking, in certain tissues such as brain microvasculature, mild tortuosity may be normal due to spatial constraints.
Number of Connected Components	This metric counts how many isolated subgraphs exist within the overall graph.	Fewer components indicate a more continuous and integrated vascular network. High component counts reflect fragmentation or incomplete anastomosis.
Largest Component Size	This refers to the number of nodes in the biggest connected subgraph.	Incomplete or poorly connected angiogenic networks will have smaller largest components, indicating disrupted or sparse growth.
Connectivity Index	The connectivity index quantifies how well-connected the network is by comparing the size of the largest connected component to the total number of nodes in the graph. It is given by: $\:Connectivity\:Index=\:\frac{{N}_{largest}}{{N}_{total}}$ A value closer to 1 indicates a highly interconnected network, while lower values suggest fragmentation or isolated regions within the network.	This measures the dominance of the main vascular network over isolated structures. High connectivity reflects effective vascular integration and maturation.
Network Density	This reflects the proportion of actual connections (edges) in the graph relative to the maximum possible number of connections. For an undirected graph with $\:N$ nodes and $\:E$ edges, network density is defined as: $\:ND=\:\frac{2E}{N(N-1)}$	Indicates how tightly packed or redundant the network is. A denser network could suggest rapid sprouting or compensatory remodeling

### Radial zone analysis (RZA)

To assess the spatial heterogeneity of the tubule network relative to the center of the image, we performed a radial zone analysis. This method segments the image into concentric circular regions centered at the geometric midpoint of the field of view, denoted as $\:({x}_{c},{y}_{c})$. Each graph node $\:i\:\in\:G$ with spatial coordinates $\:\left({x}_{i}{,y}_{i}\right)$ was assigned to a radial zone based on its Euclidean distance from the center, which we calculated as:

5 $$\:{d}_{i}=\sqrt{{({x}_{i}-{x}_{c})}^{2}+{({y}_{i}-{y}_{c})}^{2}}$$

**Fig. 3:**
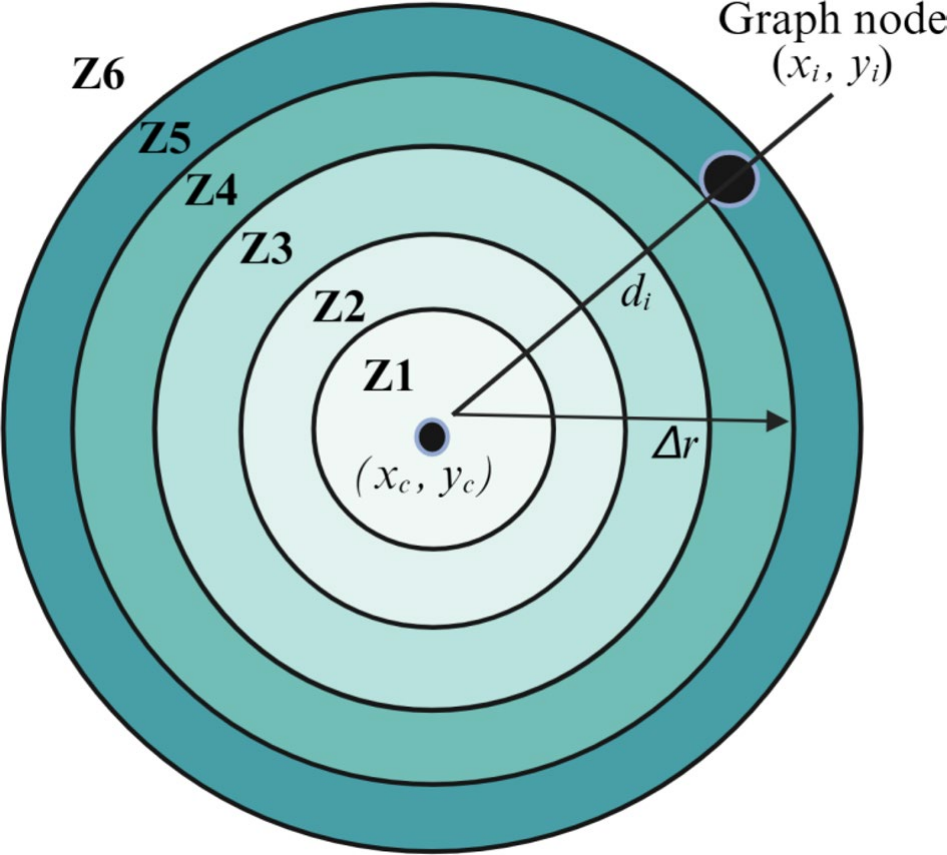
Concentric radial zones centered around the field of view, illustrating the spatial division of the angiogenic network. Each zone represents an incremental distance from the center of the image. The zones are numbered sequentially (Zone 1, Zone 2, etc.), with the distance from the center increasing as the zone number increases. This schematic shows how radial zoning was used in the pipeline to analyze the distribution of angiogenic nodes across different spatial regions of the skeletonized image

The space was partitioned into non-overlapping annular bands of fixed width $\:\varDelta\:r$ (50 pixels) and nodes were assigned to zone $\:{Z}_{k}$ where $k = \left\lfloor {{{{d_i}} \over {\Delta r}}} \right\rfloor + 1$. We defined up to six zones (for $\:k$= 1 to 6) corresponding to distances from the center up to 300 pixels (Fig. 3). Once nodes were grouped into zones, we computed the number of nodes per zone $\:{N}_{k}$, resulting in a spatial density profile where:

6 $$\:Zone\:distribution=\{{N}_{1},\:{N}_{2},\:\dots\:,{N}_{6}\}$$

However, because each successive annular zone covers a larger area than the inner ones and to ensure meaningful comparisons across zones, we normalized the number of nodes in each radial zone by the corresponding annular area, as follows:

7 $$\:{Node\:density}_{zone\:i}=\raisebox{1ex}{${Node\:count}_{zone\:i}$}\!\left/\:\!\raisebox{-1ex}{${\pi\:(r}_{outer}^{2}-{r}_{inner}^{2})$}\right.\:\:$$

Here, $\:{r}_{outer}^{2}$ and $\:{r}_{inner}^{2}$ are the outer and inner radii of the $\:{i}^{th}$annular zone.

From these values, we calculated five heterogeneity metrics that describe different aspects of spatial organization: *standard deviation*,* entropy*,* coefficient of variation (CV)*,* radial gradient and linear slope*. Standard deviation reflects the overall variability in density across zones, while entropy captures the uniformity of distribution, with higher values indicating a more even spread of vascular structures. The CV accounts for variability relative to the average density, allowing comparisons across images with differing overall network complexity. The radial gradient quantifies the difference between central (Zone 1) and peripheral (Zone 6) densities, providing a measure of center-focused versus edge-focused growth. Lastly, linear slope was derived from fitting a regression line through the zone densities, this heterogeneity metric indicates whether the vascular growth trends outward (positive slope), inward (negative slope), or is evenly spread (slope near zero). This method can explain spatial organization, network expansion, and maturation of the angiogenic network over time.

### Statistical analysis

Graph-theoretic metrics were computed for each image to quantify angiogenic network morphology. Data was analyzed using Python (v3.10.9) and key packages including SciPy, Seaborn and Scikit-learn. Some graph-based metrics scale with the number of nodes therefore these metrics were normalized by the number of nodes per image to control for scale effects and allow for direct statistical comparison across conditions. Metrics normalized by node counts are reported as average values per node across each image. Each experimental condition and time point included six biological replicates (*n* = 6). For comparisons between cell densities (sparse vs. dense), a total of nine images were analyzed per condition (*n* = 9). For temporal comparisons (2 h vs. 4 h–2 h vs. 18 h), six replicates were analyzed per time point (*n* = 6), all derived from the same cell density condition. To assess differences in network structure between experimental conditions, we applied the non-parametric Mann–Whitney U test, comparing sparse versus dense seeding densities for each metric. Effect sizes were quantified using Cliff’s Delta, with magnitudes classified as negligible (< 0.147), small (0.147–0.33), medium (0.33–0.474), and large (> 0.474) [32] For visualization, box plots were generated for each metric, with significance thresholds (*p < 0.05, **p < 0.01 and ***p < 0.001). The discriminative power of each metric was further evaluated using receiver operating characteristic area under the curve (ROC AUC) analysis, with values ≥ 0.80 considered indicative of strong group separation. Binary labels were assigned for seeding density (sparse = 0, dense = 1), and ROC-AUC values were computed using raw metric values as predictor scores. To assess temporal changes in angiogenic network topology, we performed Mann–Whitney U tests comparing 2-hour and 18-hour timepoints across all seeding densities. Corresponding AUC values were calculated to evaluate the temporal discriminability of each metric. Spearman correlation matrices were generated for each timepoint (2, 4, and 18 h.) to explore the co-dependence and temporal evolution of graph-theoretic features.

### Python implementation and data availability

The python script used for tasks described in this manuscript with microscopy images are available on Mendeley Data: https://dx.doi.org/10.17632/zjyx33c33x.1. This script facilitates the reproducibility of the framework as detailed in this study.

## Results

### Graph-theoretic framework captures and retains time-dependent angiogenic network morphology

**Fig. 4:**
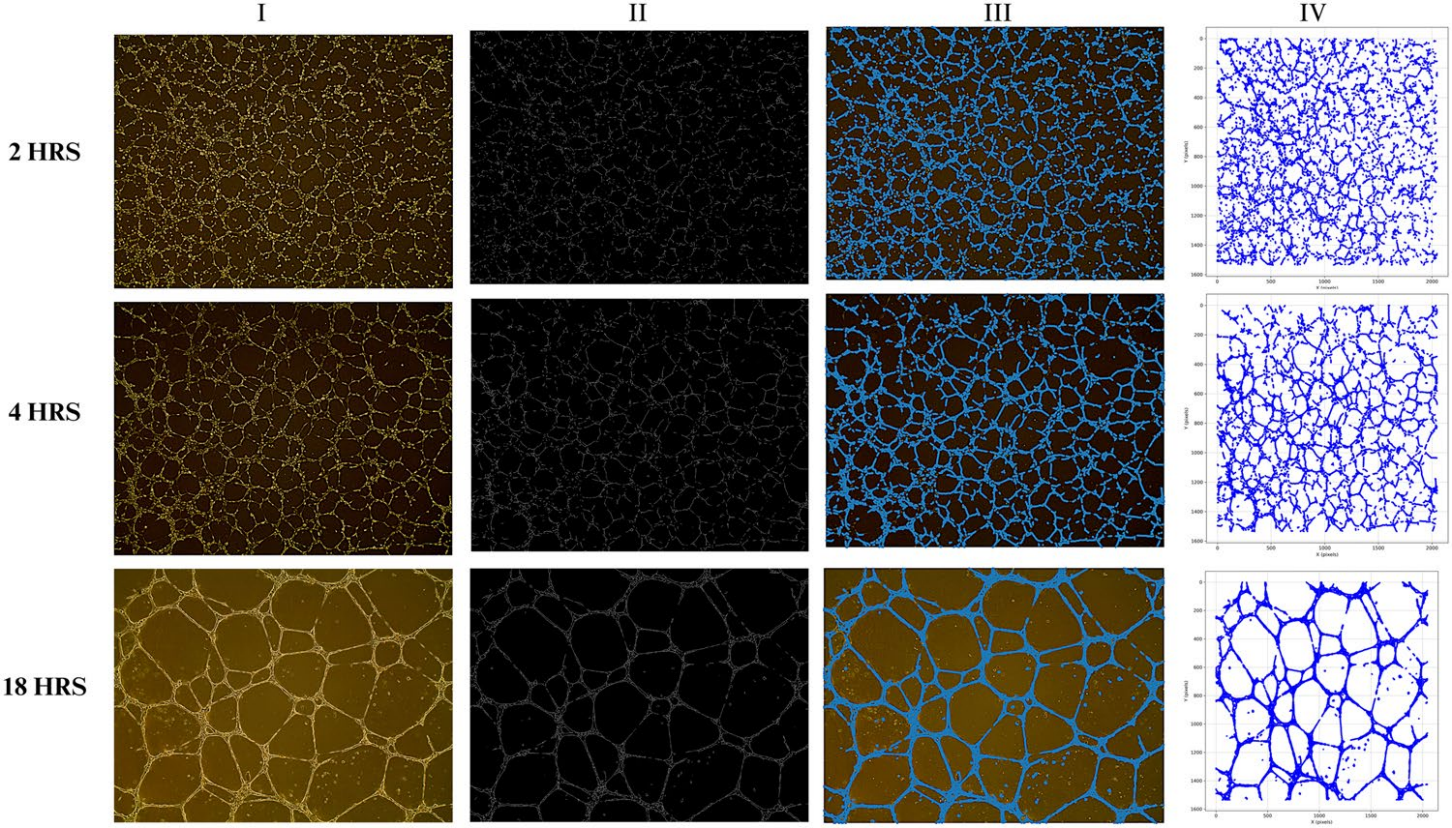
Graph-theoretic analysis of angiogenic networks at 2-, 4-, and 18-hours post-seeding (top to bottom), with columns (I–IV) showing the original brightfield image, skeletonized network, graph overlay, and 2D scatter plot of node coordinates, respectively. Each representation faithfully captures the morphological and spatial features of the original structures: tracing early branching (2 h.), transitional elongation and alignment (4 h.), and the complex, interconnected architecture of mature networks (18 h.)

To determine whether the graph-theoretic framework can capture and retain time-dependent differences in angiogenic network morphology, we applied the method to images acquired at 2, 4 and 18-hours post-seeding. For each time point, we have designed the framework to include a set of standardized outputs: the original endothelial tubule image, the skeletonized network, the overlay of the extracted graph on the image and a 2D scatter plot of the node positions. These outputs consistently reflected the expected temporal progression of network formation from sparse, disconnected tubules with minimal connectivity at 2 h to more extensive, interconnected networks with extensive graph coverage and a more uniform spatial node distribution by 18 h (Fig. 4). Visual inspection alongside quantitative graph metrics show that this approach aligns with known angiogenic dynamics and reliably preserves biologically relevant structural changes [33, 34].

### Graph metrics capture morphological shifts in angiogenic network morphology

Here we used the two distinct tube formation patterns to illustrate how this framework can be used to quantify subtle changes in network morphology that, while potentially visible, require precise characterization to evaluate therapeutic effects or early pathological deviations. Varying the cell-seeding density introduced systematic changes in the extent of cellular contact, branching complexity, and tubular connectivity.

**Fig. 5:**
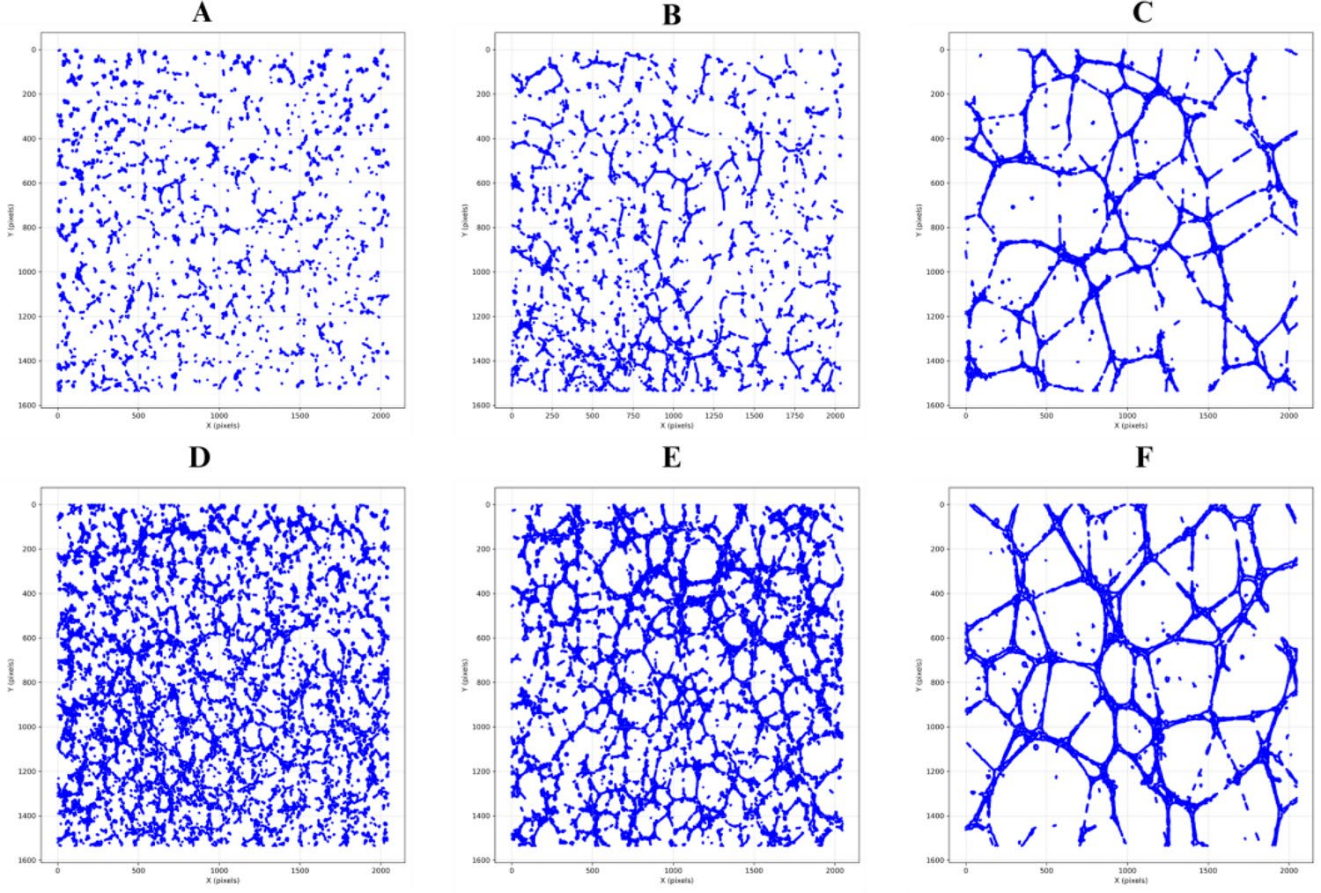
Representative tube formation 2D scatter plots of node coordinates images showing the effect of seeding density and time on angiogenic network morphology. **Top panel** (**A**–**C**): Sparse seeding conditions at 2 h. (**A**), 4 h. (**B**), and 18 h. (**C**). **Bottom panel** (**D**–**F**): Dense seeding conditions at 2 h. (**D**), 4 h. (**E**), and 18 h. (**F**). These images demonstrate that varying seeding density can be used to simulate a spectrum of angiogenic morphologies, enabling the isolation and study of underlying biological alterations

**Fig. 6:**
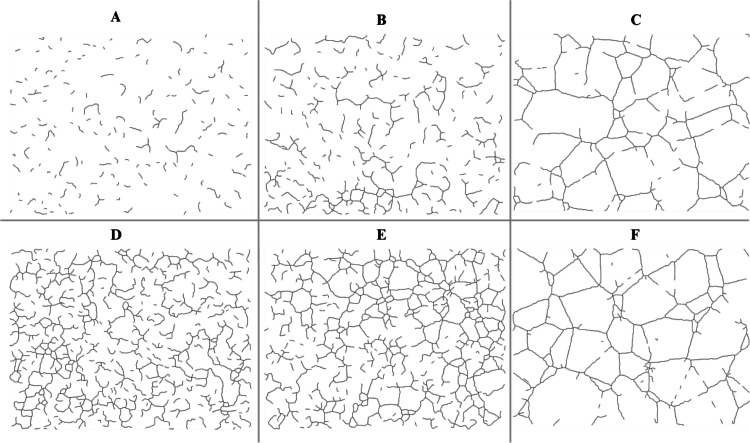
Representative segmented final binary images for same images shown in Fig. 5, analyzed using the Angiogenesis Analyzer in ImageJ. **Top row (A–C)** displays endothelial network formation under sparse seeding conditions at 2 h (**A**), 4 h (**B**), and 18 h (**C**). **Bottom row** (**D**–**F**) shows corresponding timepoints under dense seeding conditions: 2 h (**D**), 4 h (**E**), and 18 h (**F**)

Qualitative inspection of the tubule structures revealed that lower cell densities resulted in sparse, fragmented networks with fewer interconnections, whereas higher seeding densities produced dense, highly interconnected networks with pronounced nodal expansion and branching (Fig. 5). These visual differences were quantitatively validated by this framework, which consistently detected seeding-density-dependent shifts across multiple graph-derived metrics. Similarly, segmented final binary images from Angiogenesis Analyzer showed qualitative difference between both sparse and dense networks (Fig. 6).

**Fig. 7:**
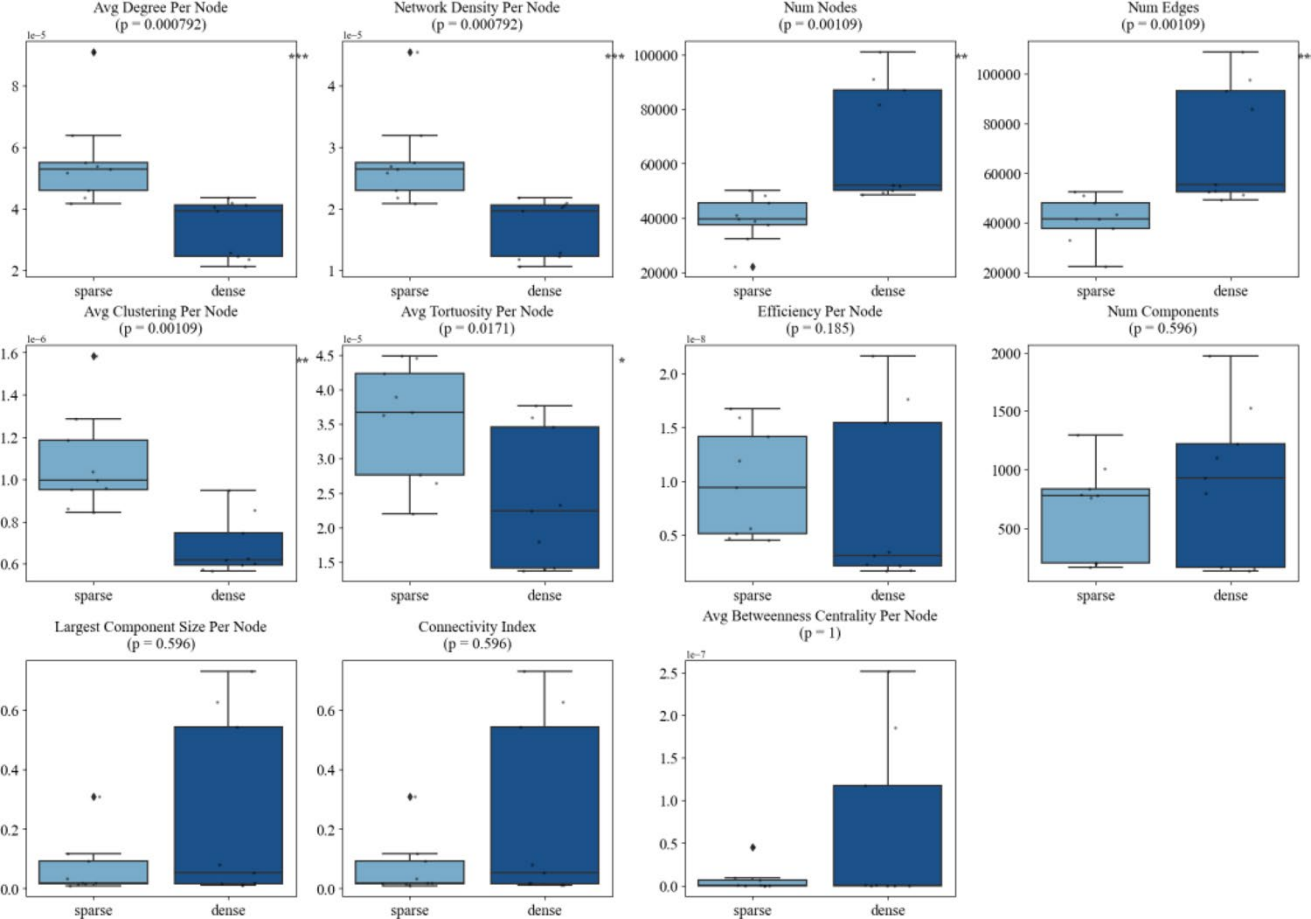
Boxplots comparing 11 graph-derived network metrics between sparse and dense seeding conditions. Significant differences (*average node degree*, *network density*, and *number of nodes*) reflect more interconnected and complex structures in dense cultures. P-values are shown above each plot, with asterisks denoting significance (*p < 0.05, **p < 0.01, ***p < 0.001). Metrics such as *average tortuosity* and *average clustering* also differentiate between network morphologies, highlighting structural variability as a result of cell-seeding density

**Fig. 8:**
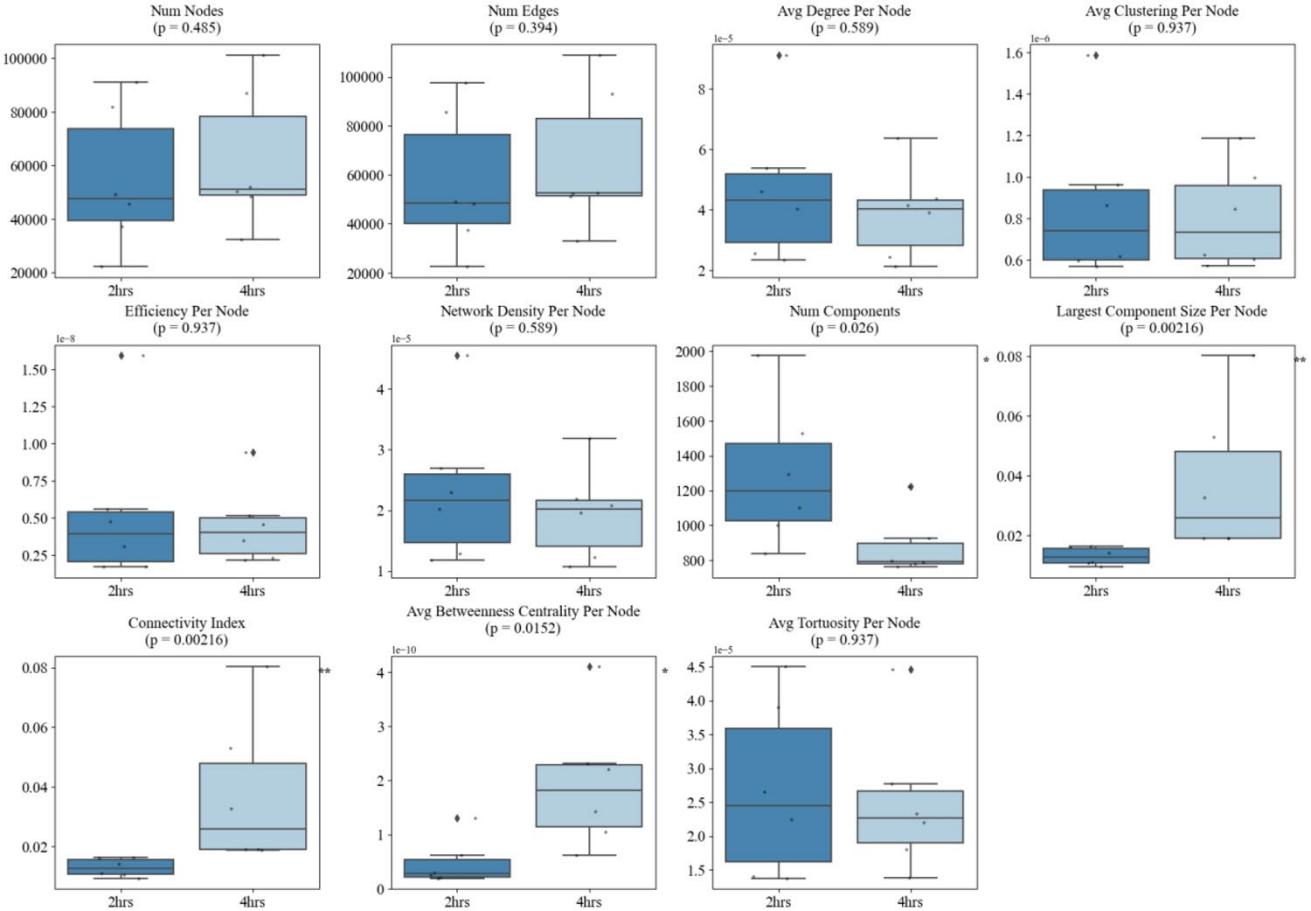
Comparison of graph-theoretical network metrics between 2-hour and 4-hour timepoints. Box plots show the distribution of 13 vascular graph metrics, highlighting significant increases in connectivity index (*p* = 0.00216), average betweenness centrality (*p* = 0.0152), and largest component size (*p* = 0.00216), alongside a reduction in number of components (*p* = 0.026). These early shifts suggest the onset of vascular network consolidation as early as 4 h

**Fig. 9:**
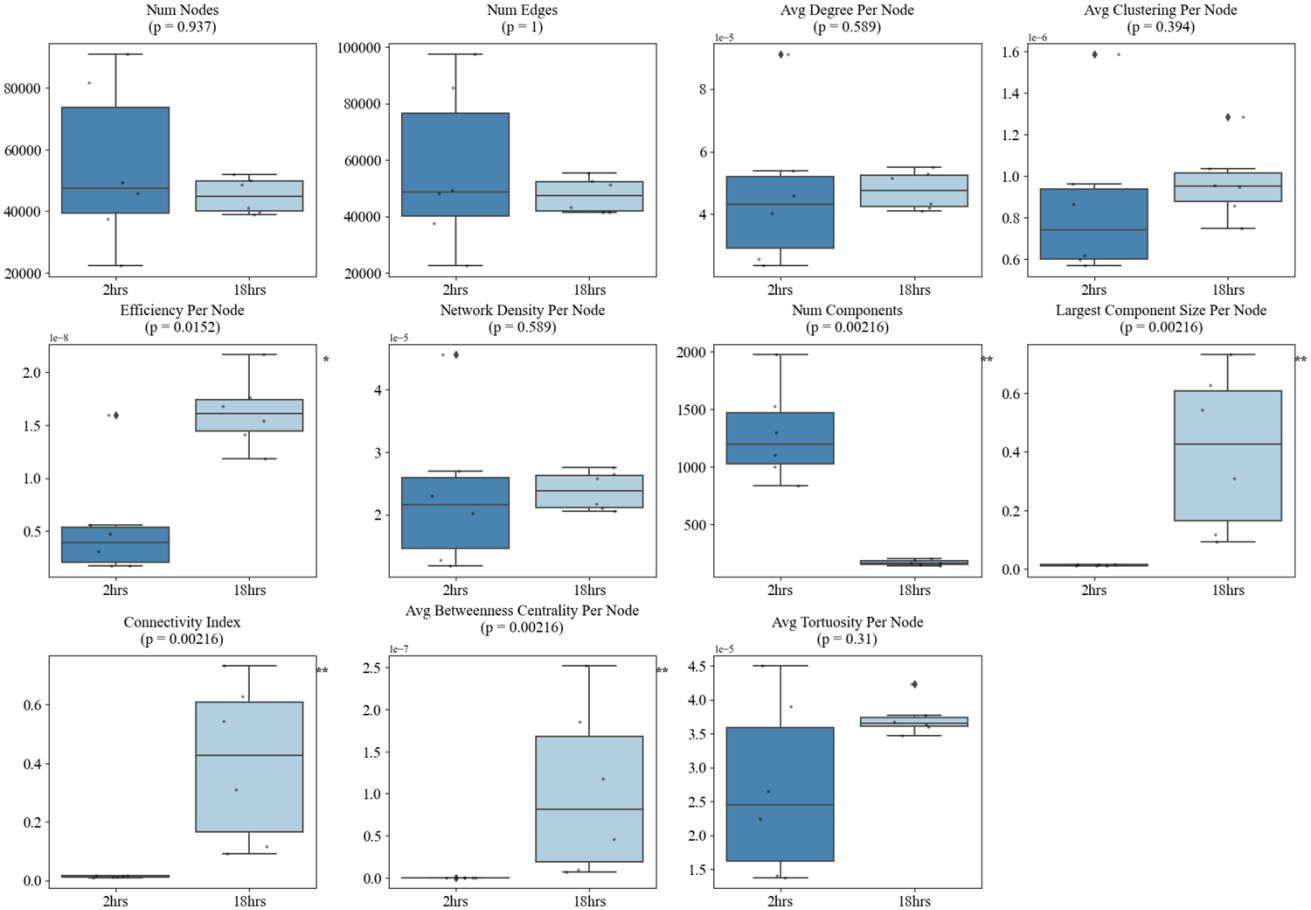
Comparison of graph metrics between 2-hour and 18-hour timepoints. Boxplots depicting the evolution of network metrics from 2 h. to 18 h. Significant increases in efficiency (*p* = 0.0152), *connectivity index* (*p* = 0.00216), *largest component size* (*p* = 0.00216), and *average betweenness centrality* (*p* = 0.00216) show progressive network integration and maturation. Metrics like number of components significantly decreased, suggesting consolidation into a dominant vascular structure

Quantitatively, our temporal and structural analyses of these angiogenic networks reveal the capability of this framework to provide detailed information to objectively quantify vascular network organization during angiogenesis. Importantly, distinct graph-based metrics were found to be differentially sensitive to either morphological phenotype (sparse vs. dense) or temporal maturation (2 h vs. 18 h). Sparse networks exhibited significantly higher *average node degree* (*p* = 0.00079), *network density* (*p* = 0.00079), *average clustering coefficient* (*p* = 0.00109), and *average tortuosity* (*p* = 0.0171), reflecting locally dense, curved, and fragmented structures. Conversely, dense networks were characterized by greater overall *number of nodes and edges* (*p* = 0.00109), indicating higher complexity (Fig. 7). Across time, the angiogenic networks evolved from fragmented architectures at 2 h into well-integrated, centralized systems by 18 h. This transition was marked by significant reductions in *number of components* (*p* = 0.00216) and increases in *largest component size* (*p* = 0.00216), *connectivity index* (*p* = 0.00216), *average betweenness centrality* (*p* = 0.00216), and *efficiency* (*p* = 0.0152). Notably, some of these hallmarks of vascular maturation such as the *number of components* (*p* = 0.026), *largest component size* (*p* = 0.026), *connectivity index* (*p* = 0.00216), and average *betweenness centrality* (*p* = 0.0152) began to diverge as early as 4 h, suggesting that structural integration is initiated well before full network consolidation at 18 h. These changes reflect the emergence of a dominant, mature angiogenic network (Figs. 8 and 9).

Interestingly, we observed that different metrics capture distinct aspects of vascular behavior: some excel at discriminating between morphologically distinct network states (sparse vs. dense), while others more effectively track consistent temporal trends during angiogenic maturation. For instance, *average node degree* (*p* = 0.00079), *network density* (*p* = 0.00079), *clustering coefficient* (*p* = 0.00109), and *tortuosity* (*p* = 0.0171) were significantly different between sparse and dense networks but did not show significant changes from 2 to 4 h (*all p* > 0.3), nor from 2 to 18 h (*all p* > 0.1). This suggests that these metrics are most sensitive to static morphological features such as how locally compact or curved the network appears rather than dynamic changes during maturation. In contrast, metrics like *efficiency* (*p* = 0.0152), *connectivity index* (*p* = 0.00216), *largest component size* (*p* = 0.00216), *number of components* (*p* = 0.00216), and *average betweenness centrality* (*p* = 0.00216) not only exhibited strong temporal trends but also began shifting significantly between 2 and 4 h (e.g., number of components *p* = 0.026; largest component size *p* = 0.00216; connectivity index *p* = 0.00216). However, these same metrics were less effective at distinguishing sparse vs. dense network morphologies (*all p* > 0.1, except for *tortuosity* and *efficiency*), showing that they primarily track longitudinal network integration rather than initial morphological variation.

**Fig. 10:**
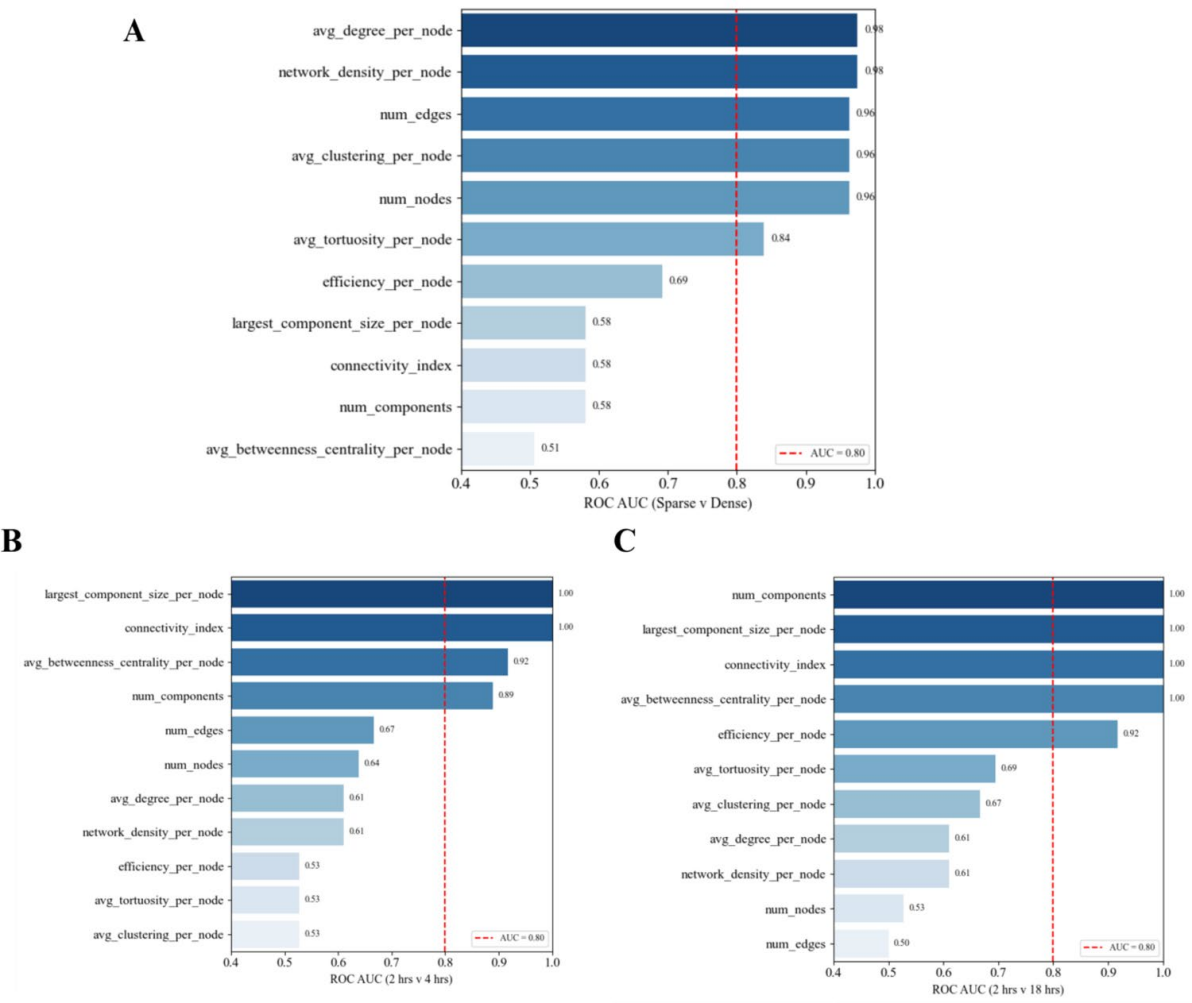
ROC-AUC of graph metrics between sparse and dense networks (**A**), 2 h v 4 h networks (**B**) and 2 h v 18 h networks. Red dashed line indicates the 0.80 AUC threshold for strong discrimination

ROC AUC (*Receiver Operating Characteristic – Area Under the Curve*) analysis confirmed that these two biological processes: morphological distinction and network maturation are best captured by different subsets of metrics. Morphology was best distinguished by *average node degree* (AUC = 0.98), *network density* (AUC = 0.98), *clustering coefficient* (AUC = 0.96), and *number of edges* (AUC = 0.96), *number of nodes* (AUC = 0.96) and *average tortuosity* (AUC = 0.84) (Fig. 10A). In contrast, *number of components*, *largest component size*, *connectivity index*, and *average betweenness centrality* each perfectly classified 2 h vs. 18 h networks (AUC = 1.00), highlighting their sensitivity to structural integration over time.

Importantly, intermediate remodeling between 2 h. and 4 h. was also detectable: *connectivity index* (AUC = 1.00), *average betweenness centrality* (AUC = 0.92), *largest component size* (AUC = 0.89), and *number of components* (AUC = 0.89) demonstrated high discriminative power (Fig. 10B), suggesting these metrics are sensitive not just to end-stage maturation, but also to early network consolidation events. Meanwhile, metrics associated with morphology performed less well in this transitional context (all AUCs < 0.69), which reinforces the idea that distinct metrics are optimized for different biological questions. Notably, depending on where the threshold is set, *efficiency* can exhibit strong performance in both contexts (AUC = 0.69 for morphology; AUC = 0.92 for end-stage maturation), showing its ability to characterize both structural and functional vascular properties (Fig. 10C).

**Fig. 11:**
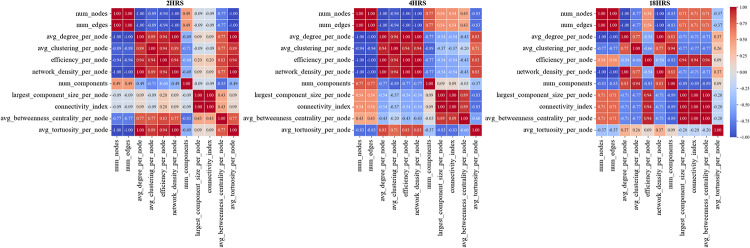
Correlation Heatmaps showing pairwise Spearman correlation coefficients among 11 graph-based network metrics extracted from tubule formation assays at 2 h (left), 4 h (middle), and 18 h (right)**(C)**. The heatmaps illustrate dynamic changes in the relationships between network properties as endothelial structures mature over time

Furthermore, Spearman correlation analysis of graph-based metrics extracted from tube formation assays at 2, 4, and 18 h revealed deeper insights into the dynamic shifts in vascular network organization (Fig. 11). At 2 h, *number of nodes and number of edges* were strongly negatively correlated with *efficiency* (ρ = –0.94), *network density* (ρ = –1.00), and *tortuosity* (ρ = –1.00), indicating a fragmented and inefficient network with many disconnected branches. By 4 h, the network began reorganizing, as *tortuosity* showed strong positive correlations with *efficiency* (ρ = +0.83), *network density* (ρ = +0.83), and *clustering coefficient* (ρ = +0.71), suggesting the emergence of looped, more organized structures. At 18 h, a mature, highly interconnected network was evident, marked by strong positive correlations among *efficiency*, *clustering coefficient*, *network density*, and *average betweenness centrality* (all ρ ≥ +0.89). Simultaneously, *number of components* was increasingly negatively correlated with *efficiency* (ρ = –0.89) and *average betweenness centrality* (ρ = –1.00), reflecting the consolidation into a unified, functionally optimized vascular structure.

**Fig. 12:**
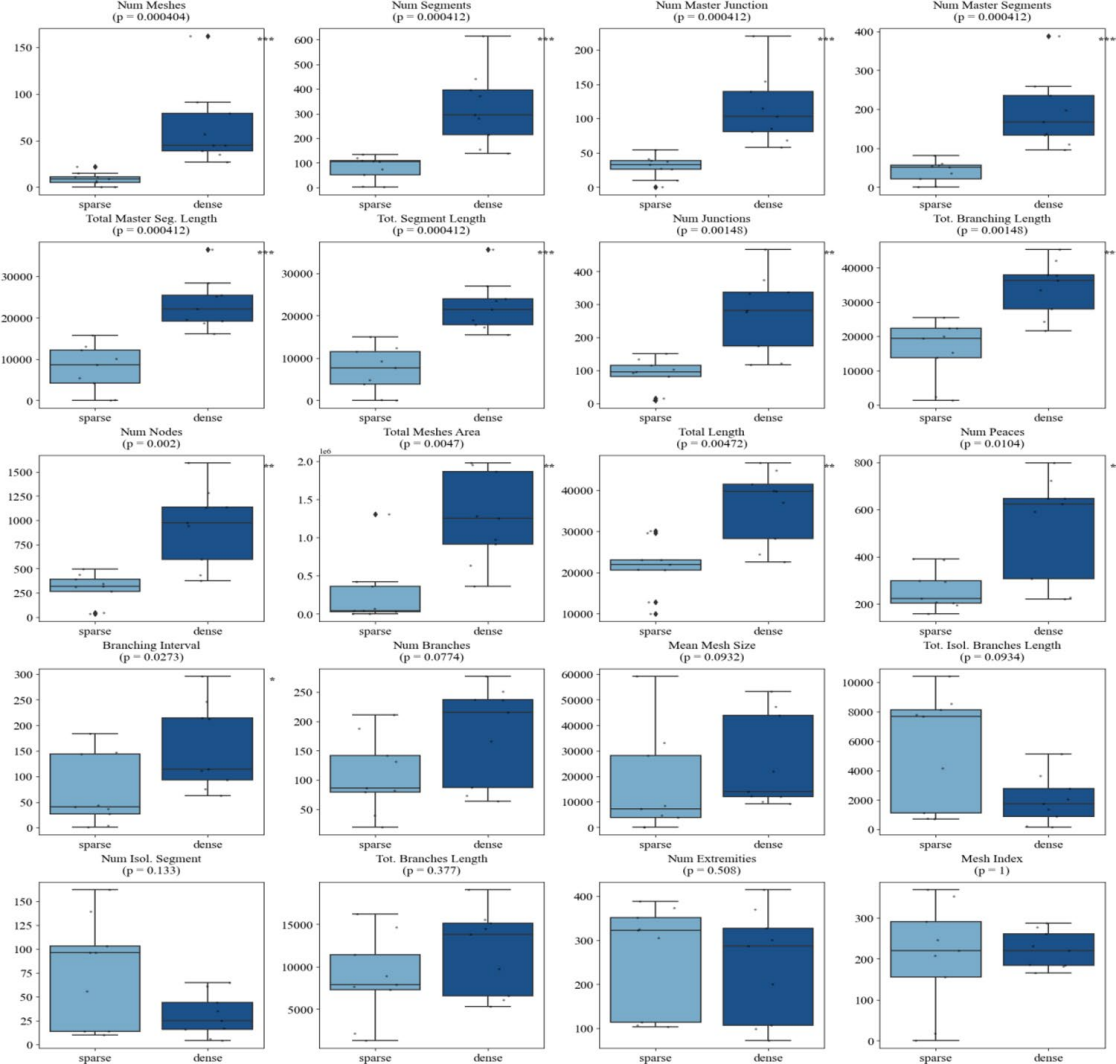
Comparative morphometric analysis of sparse vs. dense angiogenic networks using Angiogenesis Analyzer. Metrics such as *number of meshes, segments, master junctions, total segment length*, and *number of nodes* were significantly higher in dense networks, indicating enhanced vascular complexity (*p* < 0.01 for most). Others, such as *number of isolated segments* and *total branch length*, showed no significant difference (*p* > 0.1). This indicates metrics that are sensitive to morphological differences

**Fig. 13:**
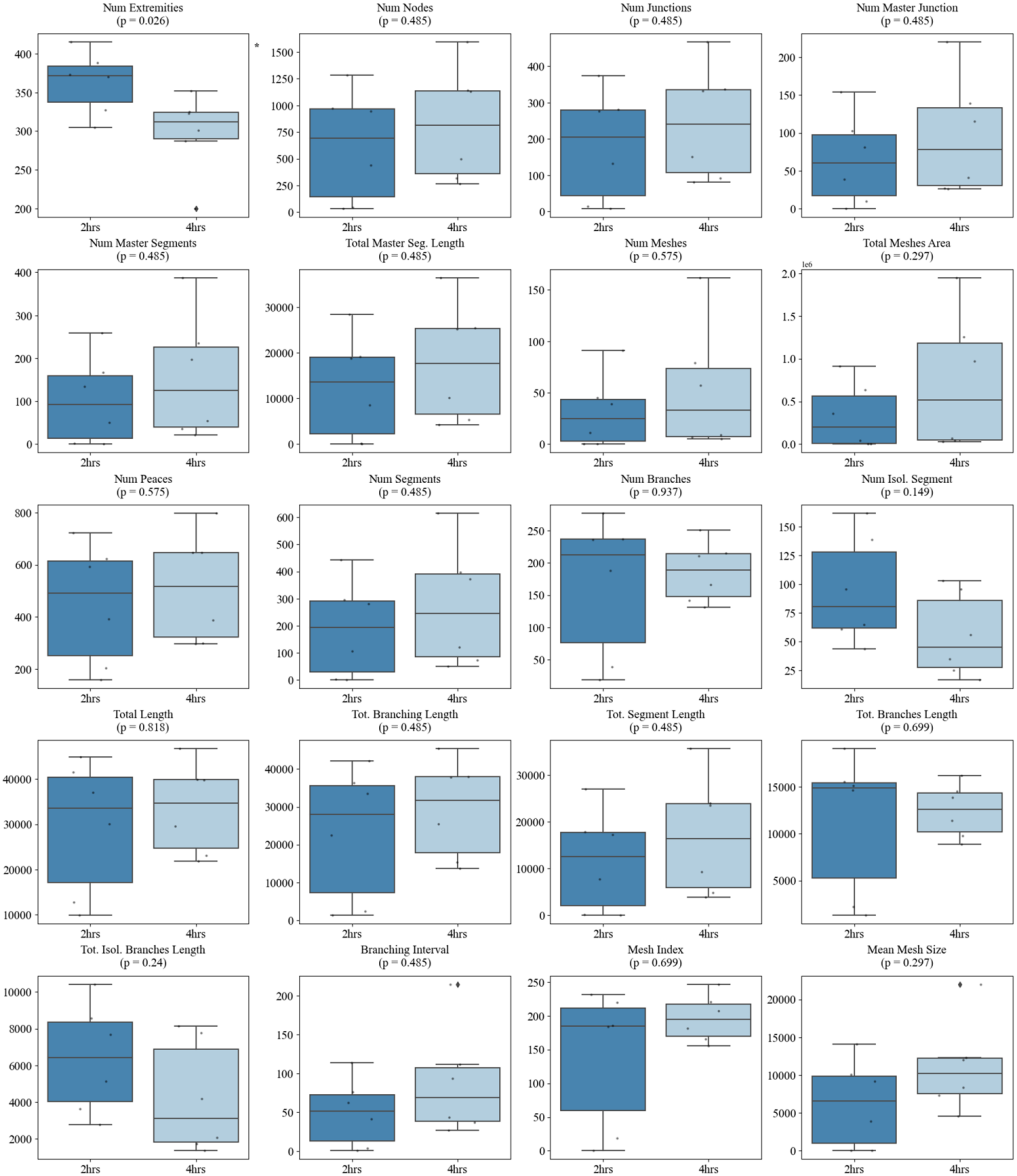
Comparison of Angiogenesis Analyzer metrics between 2-hour and 4-hour timepoints. Box plots show the distribution of 20 metrics. Most features did not differ significantly (all *p* > 0.1), except for *number of extremities* (*p* = 0.026), suggesting limited network maturation within this early phase

**Fig. 14:**
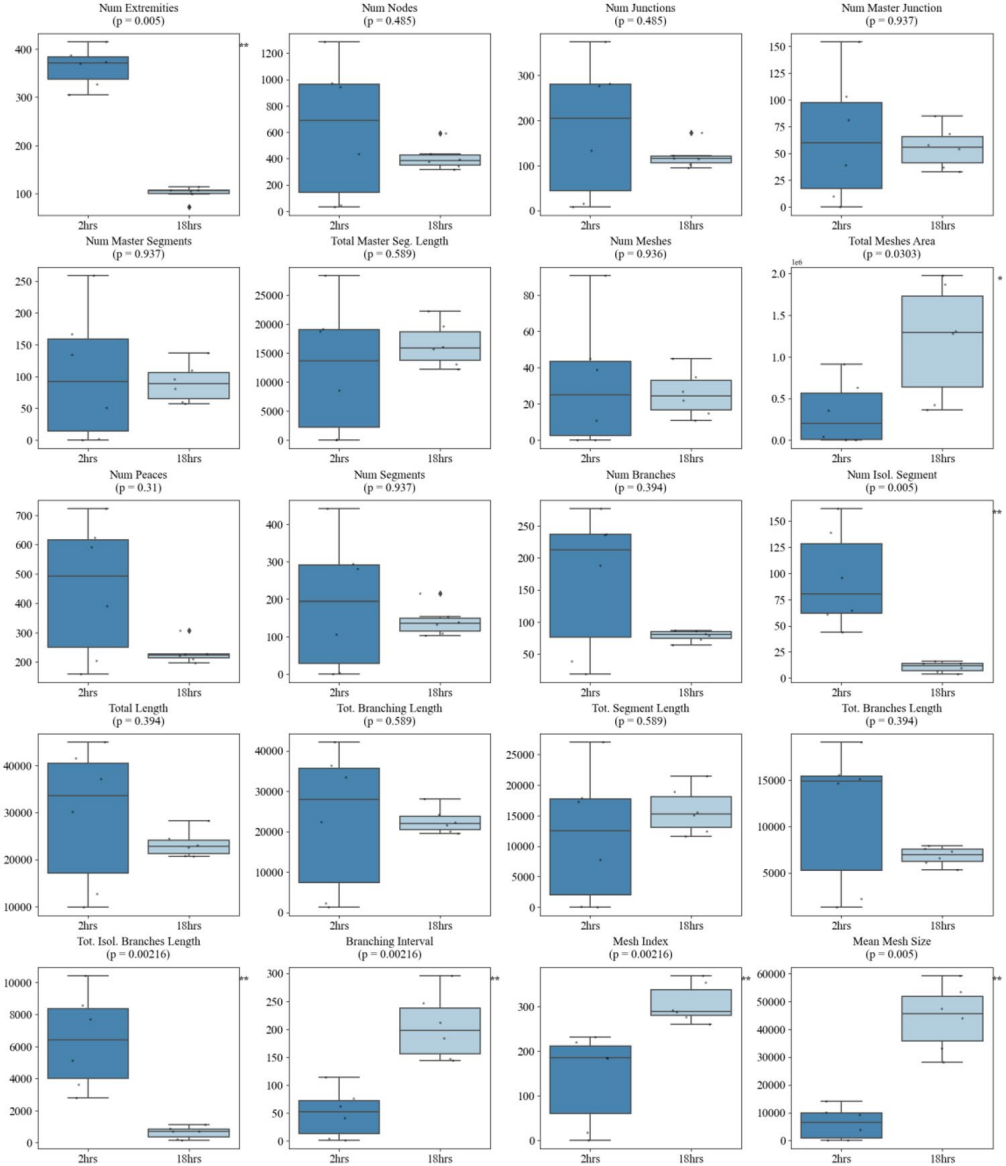
Comparison of Angiogenesis Analyzer metrics between 2-hour and 18-hour timepoints. Several features demonstrated significant temporal changes, including *mesh index* (*p* = 0.00216), *total isolated branches length* (*p* = 0.00216), *number of isolated segments* (*p* = 0.005), *mean mesh size* (*p* = 0.005), and *total meshes area* (*p* = 0.0303), reflecting network consolidation and maturation over time

Similarly, the majority of morphometric parameters extracted using the standard Angiogenesis Analyzer in ImageJ demonstrated strong discriminatory power between morphologically distinct vascular networks. Specifically, 13 out of 20 metrics significantly differentiated sparse versus dense seeding conditions, with dense networks exhibiting markedly higher values for features such as *total length*, *number of meshes*, *number of segments*, *master junctions*, *total segment length*, *number of nodes* etc. (*p* < 0.01 for most comparisons) (Fig. 12). However, when assessing dynamic changes associated with angiogenic maturation over time, the comparison between 2-hour and 4-hour sparse networks revealed minimal differences, with only the *number of extremities* showing a statistically significant change (*p* = 0.026), indicating limited early network remodeling (Fig. 13). By 18 h, additional metrics reflected significant structural maturation, including reductions in the *number of extremities* (*p* = 0.005), *number of isolated segments* (*p* = 0.005), and *total isolated branch length* (*p* = 0.00216), as well as increases in *branching interval* (*p* = 0.00216), *total mesh area* (*p* = 0.0303), *mesh index* (*p* = 0.00216), and *mean mesh size* (*p* = 0.005) (Fig. 14).

### Radial heterogeneity metrics reveal spatial patterning shifts with network morphology and time

**Fig. 15:**
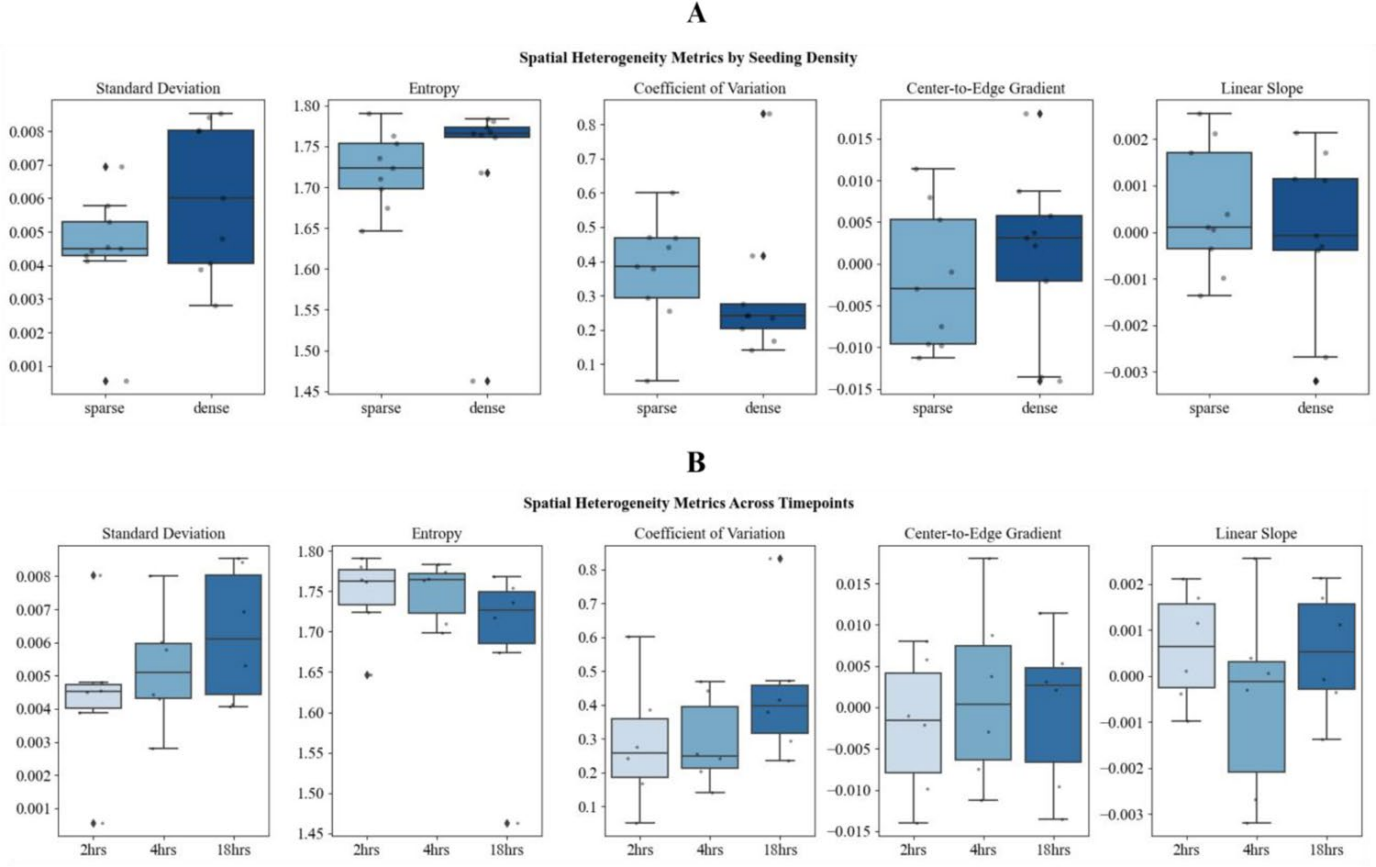
Radial spatial heterogeneity metrics by seeding density and timepoint. Boxplots showing five radial spatial heterogeneity metrics: *standard deviation, entropy, CV, center-to-edge gradient,* and *linear slope* across seeding density conditions (**A**) and timepoints (**B**). Metrics of directional growth (gradient, slope) remained near zero, suggesting isotropic radial expansion

Radial zone analysis of the 6 concentric zones shows that the dense angiogenic network exhibited higher overall variability in vascular distribution across the zones, as indicated by greater *standard deviation* (mean = 0.006, 95% CI: 0.005–0.008) compared to the sparse network (mean = 0.004, CI: 0.003–0.006). However, dense networks also showed more uniform spread across zones, with higher *entropy* (mean = 1.731, CI: 1.664–1.798 vs. 1.722, CI: 1.692–1.751). Furthermore, the sparse network demonstrated higher relative variability through a greater *coefficient of variation* (CV: 0.372, CI: 0.269–0.476 vs. 0.306, CI: 0.167–0.445), which can be interpreted as less coordinated growth across zones (Fig. 15A). Radial bias metrics which include *center-to-edge gradient* and *linear slope* remained near zero across densities, indicating largely isotropic expansion.

Over time, spatial complexity increased, with *standard deviation* rising from 2 h. (mean = 0.004, CI: 0.002–0.006) to 18 h. (mean = 0.006, CI: 0.005–0.008), while *entropy* declined slightly, suggesting increasing regional specialization. CV also rose at 18 h. (mean = 0.438, CI: 0.269–0.607), showing a trend toward heterogeneous but patterned maturation (Fig. 15B). These findings indicate that while angiogenic growth is spatially balanced, it becomes progressively more complex and compartmentalized over time and radial zone analysis can be used to observe deviations from the normal tube formation patterns.

**Table 2:** Summary of graph-based metrics based on their sensitivity to morphological vs. temporal angiogenic features

Graph Metrics	Morphology-sensitive (Sparse vs. Dense)	Temporal-sensitive (Early – 2 vs. 4 h.)	Temporal-sensitive (Late – 2 vs. 18 h)
Number of Nodes	✓	–	–
Number of Edges	✓	–	–
Average Node Degree	✓	–	–
Average Clustering Coefficient	✓	–	–
Efficiency	–	–	✓
Average betweenness centrality	–	✓	✓
Average tortuosity	✓	–	–
Number of Connected Components	–	✓	✓
Largest Component Size	–	✓	✓
Connectivity index	–	✓	✓

## Discussion

The endothelial tube formation assay is a widely used in vitro model for studying angiogenesis, yet its quantitative evaluation is sometimes limited by subjective interpretation or oversimplified metrics that fail to capture the functional complexity of vascular morphogenesis. In this study, we introduced and validated a graph-theoretic framework that enables objective, high-resolution quantification of angiogenic network morphology and spatial organization over time. This approach involves the conversion of the tubule morphology into mathematically tractable graphs. Using these graphs, it allows high-dimensional, interpretable assessment of angiogenic behavior. Traditional approaches to quantitative analysis rely heavily on pixel-based morphometrics extracted from binarized images using tools such as Angiogenesis Analyzer, MetaXpress, AngioTool and other commercial platforms [35]. While these methods effectively measure structural features such as total tubule length, node count, junction density, etc. they treat networks as collections of discrete objects, lacking the capacity to represent vascular connectivity or topology. Moreover, they are often static and insufficiently sensitive to subtle temporal or spatial changes that accompany angiogenic remodeling. The proposed graph-based method captures both local and global features of network organization, offering a more integrated and biologically meaningful representation of angiogenic structure and its evolution over time.

We have used these well-established network metrics to demonstrate that this framework reliably preserves biologically relevant network morphology at multiple time points post-seeding, reproducing well-established hallmarks of angiogenesis. At 2 h, the networks were fragmented and sparse, while by 18 h, they had matured into highly interconnected architecture. These structural transitions were readily captured in our standardized output images and graph overlays, confirming the sensitivity of the framework to dynamic remodeling. This is consistent with previous reports emphasizing the transient nature of early stage tubulogenesis, followed by stabilization and integration [36, 37].

Importantly, our use of multiple graph-derived metrics enabled nuanced characterization of the evolving angiogenic phenotype. Sparse networks displayed higher *average degree per node*, *clustering coefficient*, and *tortuosity* which are network metrics that reflect localized complexity and curved, less efficient paths. These traits align with earlier findings that suboptimal angiogenesis often manifests in disorganized, fragmented structures [38, 39]. In contrast, dense angiogenic networks demonstrated higher overall node counts, a hallmark of increased cellular participation and more complete network formation. Certain metrics (*average degree*, *clustering coefficient*) being highly sensitive to angiogenic network morphology but not time, while others (*efficiency*, *largest component size*, *betweenness centrality*) were most responsive to temporal progression, suggests that these graph parameters capture distinct biological dimensions: morphological phenotype versus maturation status (Table 2). As a result, depending on the user’s research question, an appropriate quantification metric can be selected.

ROC AUC analyses further reinforced this, revealing that metrics such as *average degree* and *clustering coefficient* were optimal for distinguishing sparse versus dense states (AUC > 0.95), while *largest component size* and *connectivity index* perfectly classified early versus late-stage networks (AUC = 1.0). These findings support the use of targeted graph metrics for specific biological questions, whether distinguishing between treatment effects that alter network morphology or identifying temporal markers of angiogenic integration.

The utility of this graph-theoretic model was further supported by Spearman correlation analyses across time points. At 2 h, the strong negative correlation between *node count* and both *efficiency* and *tortuosity* indicate inefficient, branched growth which is a hallmark of immature angiogenesis [40] By 18 h, the emergence of strong positive correlations between *efficiency*, *clustering coefficient*, and *betweenness centrality* suggests a well-integrated, centralized network capable of effective flow and communication [41, 42] Such correlations offer insights into the coordination between structural integration and functional optimization during angiogenesis.

Beyond global topology, the framework incorporates radial zone analysis to evaluate spatial heterogeneity, which is a factor often overlooked in traditional tube formation assays. Dense networks exhibiting higher entropy and lower coefficient of variation (CV) than sparse ones suggest more uniform, spatially balanced outgrowth. However, over time, CV increased while *entropy* declined slightly, indicating that maturation involves increasing regional specialization despite an overall trend toward complexity. This observation is consistent with recent studies that report spatial patterning and compartmentalization as essential features of stable vascular structures [43, 44]. The ability of our radial metrics to detect such transitions supports their utility in identifying perturbed or abnormal growth patterns, especially under pathological or therapeutic conditions.

Our analysis using the conventional method has shown that these methods provided robust quantification of gross morphometric differences but often prioritize structural endpoints and may overlook more nuanced dynamic remodeling events. These dynamic events may be important when investigating early-stage angiogenic responses to treatments, subtle phenotypic shifts during vascular maturation, or temporal disruptions in network integration under pathological conditions. As such, relying solely on conventional metrics may limit the sensitivity of angiogenesis assays, especially in studies aiming to capture transient or progressive morphogenetic changes.

Together, these results illustrate how a graph-theoretic framework can be used to detect and interpret subtle changes in angiogenic morphology that may be early indicators of therapeutic efficacy or dysfunction. This is particularly relevant for pre-clinical drug screening and mechanistic studies in which angiogenesis is modulated by treatments such as pro-angiogenic growth factors [45], extracellular vesicles [46], or plasma-based therapies [47]. The sensitivity of our approach to both structural and functional aspects of vascular formation make it a valuable tool to complement traditional methods. In addition, this framework can be beneficial for assessing cancer-associated angiogenesis, where anti-angiogenic drugs are employed to suppress tumor vascularization and hinder tumor progression.

In addition to quantifying morphological and temporal changes, the graph-theoretic framework has the potential to tailor metric selection based on the specific objectives of a given study. To guide future users, we recommend first defining the biological question which may be whether the focus is on network complexity, connectivity, spatial organization, or dynamic remodeling. For instance, researchers interested in early angiogenic sprouting or fragmentation may prioritize metrics such as *number of components*, *average node degree*, which reflect network initiation and branching behavior. For evaluating vascular maturation and integration metrics like *largest component size*, *global efficiency*, *connectivity index*, better capture the transition toward organized, functional networks. If the goal is to assess spatial heterogeneity or distribution symmetry, particularly in response to treatment or disease models, radial zone-based metrics across concentric zones can reveal subtle compartmentalization effects. We encourage users to align metric selection with hypothesized biological processes and consider multiple metrics to provide a comprehensive network characterization. This will enhance interpretability and ensure that graph-based analyses yield biologically meaningful insights.

Despite its strengths, the graph-theoretic framework presented here has several limitations. First, the segmentation step relies on Otsu’s thresholding, which, while automated and widely used, may be suboptimal in low-contrast or noisy images where vascular structures are faint or poorly defined. Although we implemented Gaussian filtering to reduce background noise, thresholding errors can still propagate through the pipeline, affecting skeletonization and graph extraction. To address this limitation, future iterations of the pipeline may benefit from integrating more adaptive and learning-based segmentation methods [48]. Second, the framework assumes a binary representation of vasculature, which may oversimplify complex 3D structures or ignore lumen size and intensity-based information, potentially omitting valuable biological detail. Third, while the extracted graph metrics are interpretable and well-established, they still are functionally ambiguous to biological context and may require complementary experimental validation to link structural patterns with functional outcomes such as perfusion or barrier integrity. Finally, although the proposed framework is computationally efficient for individual or small batches of images, high-throughput applications may face limitations because the graph construction process, particularly skeletonization and metric extraction, can become time-consuming when scaled to hundreds or thousands of images. Additionally, variability in image quality may require adaptive pre-processing, which adds further computational overhead. To enable high-throughput analysis, the framework may involve integrating parallel processing pipelines or automated quality control steps to streamline computation without compromising metric reliability.

Future work may extend this framework to in vivo imaging datasets or integrate it with machine learning models for predictive modeling. Adapting the graph-theoretic framework for in vivo applications may require integration with high-resolution 3D vascular imaging techniques such as multiphoton microscopy or micro-CT angiography. Robust segmentation algorithms that can manage motion artifacts, noise, and variable contrast in living tissues will be essential to preserve biologically relevant features. Furthermore, the computational pipeline will need to scale efficiently to process larger and more complex networks typical of in vivo datasets. Furthermore, coupling these graph-based metrics with flow simulation or other cell type interaction modeling could enhance the biological interpretability of the graph parameters. Importantly, the modular nature of this pipeline allows customization for specific applications, including diabetic angiopathy, tumor angiogenesis, or ischemia-induced remodeling.

## Conclusion

This study introduces a graph-theoretic framework as a quantitative approach for analyzing angiogenic network morphology in tube formation assays. By modeling endothelial tubule structures as mathematical graphs, we demonstrate the ability to detect and interpret both morphological differences and temporal maturation in vascular networks. Our analysis reveals that specific graph metrics, such as *average node degree*, *clustering coefficient*, and *network density*, among others are sensitive to morphological variations, while metrics like *connectivity index*, *component size*, and *betweenness centrality* effectively capture dynamic network integration over time. Additionally, radial heterogeneity analysis uncovers subtle shifts in spatial organization during angiogenic progression. Together, these findings reveal the biological relevance and analytical rigor of graph-based metrics which offers a scalable and objective method to complement traditional tube formation quantification. This framework holds promise for enhancing the sensitivity of preclinical angiogenesis studies, facilitating therapeutic screening, and advancing our understanding of vascular patterning in both health and disease.

## Supplementary Information

Below is the link to the electronic supplementary material.

Supplementary Material 1

## Data Availability

No datasets were generated or analysed during the current study.
